# Interleukin-4 gene polymorphism (C33T) and the risk of the asthma: a meta-analysis based on 24 publications

**DOI:** 10.1186/s12881-020-01169-w

**Published:** 2020-11-23

**Authors:** Danyal Imani, Mohammad Masoud Eslami, Gholamreza Anani-Sarab, Mansur Aliyu, Bahman Razi, Ramazan Rezaei

**Affiliations:** 1 Department of Immunology, School of Public Health, Tehran University of Medical Sciences (TUMS), Tehran, Iran; 2 Department of Hematology, School of Medicine, Tarbiat Modares University (TMU), Tehran, Iran; 3 Cellular and Molecular Research Center, Birjand University of Medical Sciences, Birjnad, Iran; 4 Department of Medical Microbiology and Parasitology, Faculty of Clinical Sciences, College of Health Science, Bayero University, Kano, Nigeria; 5 Department of Immunology, School of Medicine, Shahid Beheshti University of Medical Sciences, Tehran, Iran

**Keywords:** IL-4, Polymorphism, Interleukin- 4, Meta –analysis, Asthma

## Abstract

**Background:**

Previous studies evaluated the association of IL-4 C33T polymorphism and risk of bronchial asthma but failed to establish a consistent conclusive association. In the present meta-analysis, we intend to define a more reliable estimate of the association in the presence of filling published literature.

**Methods:**

An exhaustive search in Web of Science, Scopus, and PubMed databases was performed to identify all relevant publications before September 2020, and 24 publications (28 studies) with 6587 cases and 8408 controls were included in final analysis. The association between polymorphism and risk of asthma were measured by Odd ratios (ORs) and 95% confidence intervals (CIs). Moreover, Cochran’s Q and the I^2^ statistics were used to evaluate the degree of heterogeneity between studies.

**Results:**

In the overall study populations, a significant positive association was detected under all genotype models and announced the IL-4 C33T polymorphism as a potential risk factor in the pathogenesis of asthma. In the subgroup analysis by age, a significant association between IL-4 C33T polymorphism and risk of asthma in different age groups was identified in allelic model, which highlighted the predisposing role of the T allele for the asthma risk in all three age groups. Furthermore, the results of subgroup analysis by continent were heterogenous. Accordingly, IL-4 C33T polymorphism was a risk factor in Europeans (all models except heterozygote comparison), Americans (all models except recessive and homozygote comparison) and Asians (just recessive and allelic model). Finally, the ethnicity-specific analysis disclosed a significant association between IL-4 C33T polymorphism and asthma risk in Caucasians (all genotype models except heterozygote comparison), while this association was not significant in African-Americans.

**Conclusions:**

This study suggests that IL-4 C33T polymorphism potentially acts as a risk factor for asthma in different ethnicities and age groups.

## Background

Asthma is a chronic, complex respiratory disorder in which allergen-triggered inflammatory reactions in the airways contribute to the development of symptoms, including breathlessness, cough, wheezing, and dyspnea. It has been estimated that asthma affect about 300 million people in the world [1]. Prognostic markers to detect high-risk individuals are urgently required for early identification and preventive attention. In the scientific community, genetic vulnerability to asthma is one of the main research interests [2]. In the recent decade, many studies have been focused to elucidate the susceptibility genes of asthma and several single nucleotide polymorphisms (SNPs) in these genes have been described to be related with asthma risk in different populations [3, 4]. Among different genes, interleukin 4 (IL-4) gene has been comprehensively investigated [5, 6]. IL-4 plays a major function in isotype class switching of B cells to IgE production, type 2 immune responses, and it is involved in recruitment of mast cell [7, 8]. It has thus been proposed that IL-4 may have an imperative role in the development and persistent of asthma [7]. IL-4 gene is located on long arm of chromosome 5 (5q31), a region that has been associated with asthma or related disorders such as bronchial hyper responsiveness (BHR) and atopy [9]. The IL-4 C33T single nucleotide polymorphism (rs2070874) which is located on the untranslated region (UTR) has been represented to be linked with elevated serum IgE levels and risk of asthma [10–12]. There are several studies in which association between IL-4 C33T polymorphism and asthma risk have been evaluated [5, 6, 10–31]. Nevertheless, this association remains inconsistent and inconclusive in several studies. Probably, this could be because of the small samples size examined in these studies and the small effect size of the polymorphism that failed to provide sufficient statistical power to identify statically significant associations. Accordingly, we conducted a meta-analysis to conclude a more exact estimation of the relation between the IL-4 C33T polymorphism and risk of asthma.

## Methods

We carried out this meta-analysis by following the Preferred Reporting Items for Systematic reviews and Meta-Analyses (PRISMA) statement [32]. Since our study did not contain any experimental procedure on humans and animals, thus no ethics committee confirmation was applicable.

### Search strategy

A comprehensive systematic search was applied through three major databases (MEDLINE, web of science, and Scopus) to find all potential publications considering the association between IL-4 C33T polymorphism and asthma risk released before September 2020. We searched (“asthma” [Mesh] OR “asthmatic”) AND (“interleukin-4” OR “IL-4” OR “interleukin 4”) AND (“single nucleotide polymorphism” OR “SNP” OR “polymorphisms” OR “mutation” OR “variation”), as main key words. Besides, cross reference check of review studies were screened for additional relevant papers.

### Inclusion and exclusion criteria

Initial search strategy yield 1873 studies that exported to Endnote X8. The duplicate studies which were common among databases were removed and title and abstract of remain studies were reviewed by two investigators. In cases that we could not categorize retrieved studies by title and abstract, full-text verification was performed. Eventually, studies considered eligible if met the following criteria: 1) publications that evaluate the association between IL-4 C33T polymorphism and the risk of asthma; 2) publications with extractable data to estimate odds ratios (ORs) and 95% confidence intervals; 3) publications that report genotype or allele distributions of case and controls. Duplicate articles, review articles, editorials, case reports, book chapters, republished data, comments, and studies with insufficient data after contacting with authors were all excluded. The usage of these criteria results in 24 eligible paper for the meta-analysis.

### Data extraction and quality assessment

Two researchers independently and according to a standard checklist extracted requisite data including: the first author family, country of origin, ethnicity, number of subjects in the case and the control groups for each gender, mean or range of age, applied genotyping method, distribution of alleles in cases and controls, journal and year of publication. The methodological quality of included study was scored using the Newcastle-Ottawa Scale (NOS) criteria [33]. Accordingly, publications with scores 0–3, 4–6 or 7–9 were low, moderate or high-quality, respectively.

### Statistical analysis

All data analysis was accomplished using SPSS (version 23; Chicago, IL, USA) and Stata (version 14; Stata Corporation, College Station, TX) softwares. The strength of association between SNP and the risk of asthma was measured via Odd ratios (ORs) and 95% confidence intervals (CIs). Moreover, the degree of heterogeneity between studies was assessed by the Q test (Q-statistic *P* value> 0.10, no heterogeneity vs. Q-statistic *P* value< 0.10, significant heterogeneity) and the I^2^ test (I^2^ < 25%, no heterogeneity; I^2^ = 25–50%, moderate heterogeneity; I^2^ = 50–75%, large heterogeneity, I^2^ > 75%, extreme heterogeneity) [34]. In the presence of heterogeneity random effect model (REM) should be used. Otherwise, fixed effect model (FEM) should be applied [35]. To estimate whether the results were substantially changed by any individual study, sensitivity analysis by sequential omitting of each included study was performed. Furthermore, publication bias was investigated, using Begg’s and Egger’s tests along with visual examination of the funnel plot (*p* value< 0.05 considered statistically significant) [36].

## Results

### Study characteristics

**Fig. 1:**
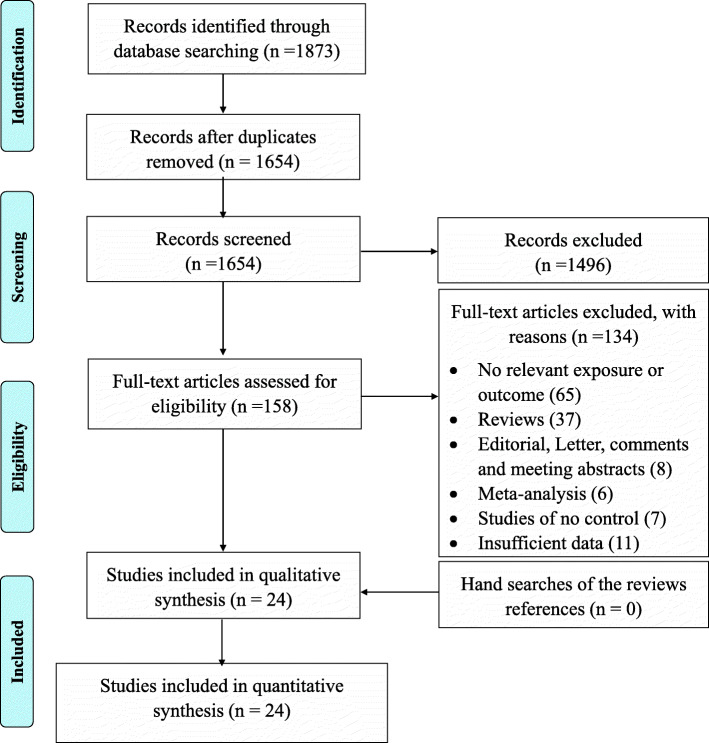
Flow diagram of study selection process

A total of 1873 articles were identified through the systematic literature search of databases. After excluding 219 duplicate studies and removing 1496 irrelevant publications based on titles/abstracts, 158 studies went under full-text screening. Of which, 134 paper were excluded. Finally, twenty-four studies qualified for quantitative analysis Fig. 1. All included studies were performed between 2000 to 2016 and had good methodological score ranging 5 to 8. Case-control design was common between eligible studies and different genotyping method were used by included studies. Tables 1 and 2 summarized the characteristics and allele distribution, genotype frequency of the eligible studies.

**Table 1:** Characteristics of studies included in meta-analysis of overall asthma

Study author	Year	Country	continent	Ethnicity	Sex cases/controls	Total cases/control	Age case/control	Genotyping method	Quality score
Suzuki et al.	2000	Japan	Asian	Caucasian	M = 50/60 F = 100/150	120 / 120	Adult	PCR-RFLP	**6**
Beghe et al.	2003	UK	European	Caucasian	M = 88/99 F = 93/89	187 / 182	Adult	PCR-RFLP	**7**
Basehore et al. *(i)*	2004	USA	American (USA white)	Caucasian	M = 93/140 F = 98/147	233 / 245	Adult	PCR	**7**
Basehore et al.*(ii)*	2004	USA	American	American- African	M = 77/91 F = 121/148	168 / 269	Adult	PCR	**6**
Basehore et al.*(iii)*	2004	USA	American (USA Hispanic)	Caucasian (Hispanic)	M = 54/62 F = 41/89	116 / 130	Adult	PCR	**6**
Park et al.	2004	Korea	Asian	Caucasian	M = 248/302 F = 85/86	532 / 170	Mixed	SnaP shot	**8**
Donfack et al. *(i)*	2005	USA	American	Caucasian	M = NR F=NR	126 / 205	Mixed	LAS	**6**
Donfack et al.*(ii)*	2005	USA	American	American- African	M = NR F=NR	205 / 183	Mixed	LAS	**6**
Garcia et al.	2005	Spain	European	Caucasian	M = NR F=NR	133 / 79	Mixed	TaqMan	**6**
Battle et al.	2007	USA	American	American- African	M = 105/156 F = 67/109	261 / 176	Mixed	PCR-RFLP	**6**
Amirzargar et al.	2009	Iran	Asian	Caucasian	M = NR F=NR	59 / 139	Mixed	PCR-SSP	**5**
Jiang et al.	2009	China	Asian	Caucasian	M = NR F = NR	24 / 24	Adult	PCR-RFLP	**5**
Daley et al.	2009	Australia	Australian	Caucasian	M = NR F = NR	643 / 751	Mixed	Illumina Bead array system	**8**
Haller et al.	2009	USA	American	American- African	M = NR F=NR	72 / 70	Adult	PCR-RFLP	**6**
Wang et al.	2009	Taiwan	Asian	Caucasian	M = 299/147 F = 245/265	446 / 510	Children	TaqMan	**7**
Berce et al.	2010	Slovenia	European	Caucasian	M = NR F=NR	106 / 89	Children	PCR-RFLP	**6**
Undarmaa et al. *(i)*	2010	Japan	Asian	Caucasian	M = NR F=NR	324 / 336	Children	TaqMan-ASA	**7**
Undarmaa et al.*(ii)*	2010	Japan	Asian	Caucasian	M = NR F=NR	367 / 676	Adult	TaqMan-ASA	**8**
Wu et al.	2010	China	Asian	Caucasian	M = 138/114 F = 118/109	252 / 227	Children	PCR-RFLP	**7**
Michel et al.	2010	Germany	European	Caucasian	M = NR F=NR	703 / 658	Children	Illumina Sentrix Bead chip	**8**
Huang et al.	2011	China	Asian	Caucasian	M = 51/49 F = 70/52	100 / 122	Children	PCR-RFLP	**6**
Yang et al.	2011	China	Asian	Caucasian	M = 101/101 F = 155/50	202 / 205	Adult	MALDI-TOF	**6**
Chen et al.	2011	China	Asian	Caucasian	M = NR F=NR	202 / 191	Children	MALDI-TOF	**7**
Micheal et al.	2013	Pakistan	Asian	Caucasian	M = NR F=NR	108 / 120	Mixed	PCR-RFLP	**6**
Miyake et al.	2013	Japan	Asian	Caucasian	M = 0/89 F = 0/1281	89 / 1281	Adult	TaqMan	**6**
Davoodi et al.	2013	India	Asian	Caucasian	M = 45/55 F = 21/29	100 / 50	Adult	Mass Array	**5**
Wang et al.	2015	China	Asian	Caucasian	M = NR F=NR	392 / 849	Children	Mass Array	**8**
Li et al.	2016	China	Asian	Caucasian	M = 134/183 F = 151/200	317 / 351	Children	PCR-RFLP	**7**

*NR* not reported, *M* male, *F* female

**Table 2:** Distribution of genotype and allele among asthma patients and controls

Study author	Asthma cases					Healthy control					P-HWE	MAF
	CC	CT	TT	C	T	CC	CT	TT	C	T		
Suzuki et al.	11	56	53	78	162	10	59	51	79	161	0/21	0/67
Beghe et al.	140	41	6	321	53	132	48	2	312	52	0/29	0/142
Basehore et al. *(i)*	153	72	8	378	88	185	56	4	426	64	0/91	0/13
Basehore et al.*(ii)*	51	83	34	185	151	87	132	50	306	232	0/99	0/431
Basehore et al.*(iii)*	48	53	15	149	83	60	57	13	177	83	0/92	0/319
Park et al.	19	164	349	202	862	7	57	106	71	269	0/84	0/791
Donfack et al. *(i)*	83	37	6	203	49	150	50	5	350	60	0/73	0/146
Donfack et al.*(ii)*	68	107	30	243	167	70	86	27	226	140	0/94	0/382
Garcia et al.	93	39	1	225	41	64	15	0	143	15	0/35	0/094
Battle et al.	85	128	48	298	224	57	87	32	201	151	0/9	0/428
Amirzargar et al.	2	56	1	60	58	61	78	0	200	78	< 0.001	0/28
Jiang et al.	0	9	15	9	39	2	10	12	14	34	0/96	0/708
Daley et al.	481	150	12	1112	174	555	181	15	1291	211	0/95	0/14
Haller et al.	21	36	15	78	66	27	33	10	87	53	0/98	0/378
Wang et al.	22	147	277	191	701	16	186	308	218	802	0/05	0/786
Berce et al.	67	31	8	165	47	51	35	3	137	41	0/3	0/23
Undarmaa et al. *(i)*	27	142	155	196	452	37	144	155	218	454	0/68	0/675
Undarmaa et al.*(ii)*	28	154	185	210	524	64	286	326	414	938	0/91	0/693
Wu et al.	6	83	163	95	409	11	87	129	109	345	0/44	0/759
Michel et al.	458	210	35	1126	280	474	173	11	1121	195	0/28	0/148
Huang et al.	1	23	76	25	175	3	49	70	55	189	0/09	0/774
Yang et al.	14	56	132	84	320	7	67	131	81	329	0/65	0/802
Chen et al.	6	72	124	84	320	6	62	123	74	308	0/58	0/806
Micheal et al.	77	31	0	185	31	93	27	0	213	27	0/16	0/112
Miyake et al.	12	33	44	57	121	160	604	517	924	1638	0/42	0/639
Davoodi et al.	65	31	4	161	39	36	14	0	86	14	0/24	0/14
Wang et al.	49	176	167	274	510	102	410	337	614	1084	0/18	0/638
Li et al.	147	170	0	464	170	170	181	0	521	181	< 0.001	0/257

P-HWE, *p*-value for Hardy–Weinberg equilibrium; MAF, minor allele frequency of control group

### Meta-analysis of IL-4 C33T polymorphism and the risk of asthma

**Fig. 2:**
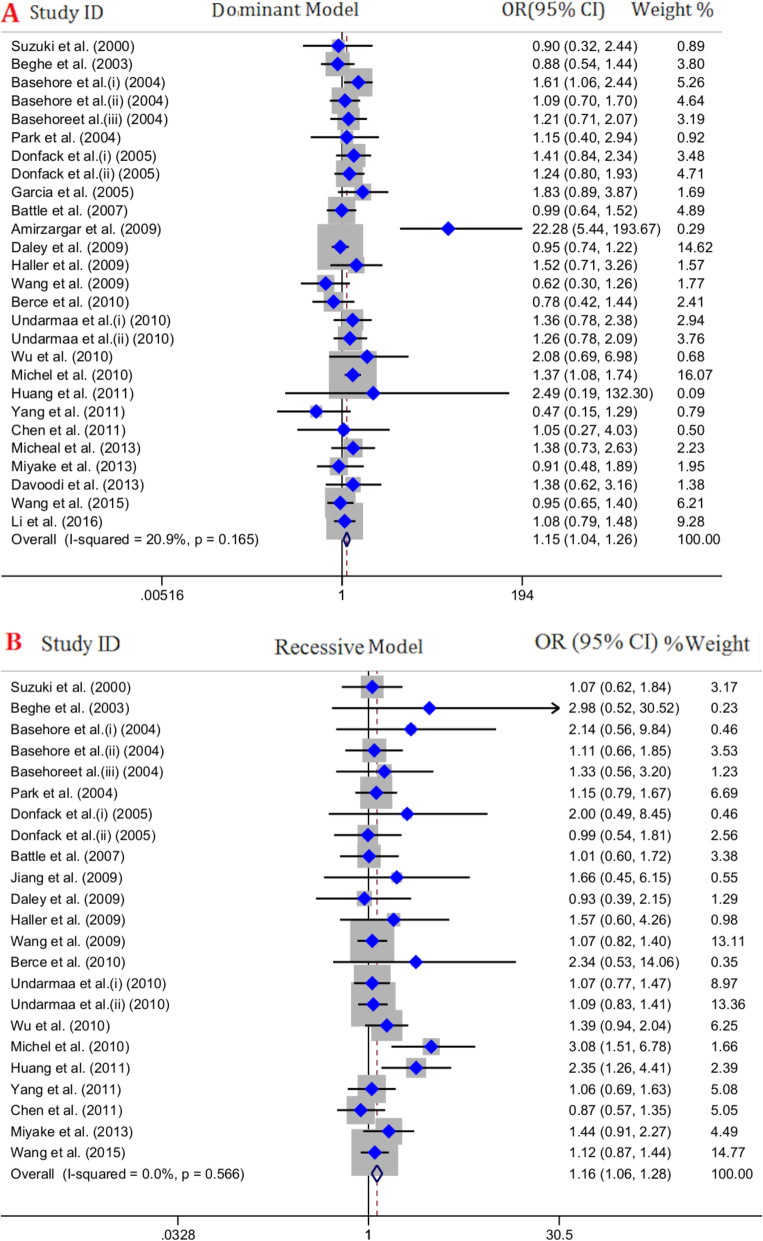
Pooled odds ratio (OR) and 95% confidence interval of individual studies and pooled data for the association between IL-4 C33T gene polymorphism and asthma risk in overall populations for **a** dominant model, **b** recessive model

Twenty-four studies with 6587 cases and 8408 healthy controls were included in final meta-analysis of overall population. Of them, 15 publications were carried out in Asian countries, 5 publications were in American countries and 4 publications were in Europe. The pooled OR indicated that IL-4 C33T polymorphism increase risk of asthma across all genotype models including dominant model (OR = 1.15, 95% CI = 1.04–1.26, *P* = ≤0.001, FEM), recessive model (OR = 1.16, 95% CI = 1.06–1.28, *P* = ≤0.001, FEM), allelic model (OR = 1.14, 95% CI = 1.07–1.21, *P* = ≤0.001, FEM), CC vs. TT model (OR = 1.21, 95% CI = 1.02–1.43, *P* = 0.02, FEM) and CT vs. TT model (OR = 1.10, 95% CI = 1–1.22, *P* = 0.05, FEM) Fig.2. The detailed findings for different analysis models are shown in Table 3.

**Table 3:** Main results of pooled ORs in meta-analysis of IL-4 (C33T) gene polymorphisms

Subgroup		Sample size	Test of association		Test of heterogeneity		Test of publication bias (Begg’s test)		Test of publication bias (Egger’s test)	
	Genetic model	Case/Control	OR	95% CI (***p***-value)	I2 (%)	P	z	P	t	P
**Overall**	Dominant model	6587 / 8404	**1.15**	**1.04–1.26 (≤0.001)**	20.9	0.16	0.77	0.44	0.90	0.37
	Recessive model	6587 / 8404	**1.16**	**1.06–1.28 ((≤0.001)**	0	0.56	2.68	0.001	2.88	0.001
	Allelic model	6587 / 8404	**1.14**	**1.07–1.21 (≤0.001)**	27.8	0.08	2.53	0.01	2.39	0.02
	CC vs. TT	6587 / 8404	**1.21**	**1.02–1.43 (0.02)**	0	0.58	1.78	0.07	1.57	0.13
	CT vs. TT	6587 / 8404	**1.10**	**1–1.22 (0.05)**	20.5	0.17	0.23	0.81	0.56	0.58
**Age subgroups**										
**Adults**	Dominant model	1678 / 3252	1.16	0.97–1.40 (0.10)	0	0.54	−0.98	0.32	−1.43	0.19
	Recessive model	1678 / 3252	1.17	0.99–1.39 (0.06)	0	0.93	2.41	0.01	3.57	0.007
	Allelic model	1678 / 3252	**1.14**	**1.02–1.26 (0.02)**	0	0.75	1.01	0.31	1.44	0.18
	CC vs. TT	1678 / 3252	1.22	0.93–1.61 (0.15)	0	0.72	0.83	0.40	0.64	0.54
	CT vs. TT	1678 / 3252	1.09	0.90–1.32 (0.91)	0.2	0.43	−1.16	0.24	−1.72	0.12
**Mixed**	Dominant model	2067 / 1823	1.14	0.96–1.34 (0.13)	55.8	0.02	2.23	0.02	3.47	0.01
	Recessive model	2067 / 1823	1.08	0.84–1.40 (0.53)	0	0.89	−0.49	0.62	0.47	0.67
	Allelic model	2067 / 1823	**1.14**	**1.01–1.29 (0.03)**	56.9	0.02	2.72	0.007	3.11	0.02
	CC vs. TT	2067 / 1823	1.10	0.77–1.57 (0.60)	0	0.88	1.47	0.14	2.01	0.13
	CT vs. TT	2067 / 1823	1.13	0.95–1.35 (0.15)	53.7	0.03	2.47	0.01	3.38	0.01
**Children**	Dominant model	2842 / 3333	1.15	0.99–1.34 (0.07)	12.4	0.33	−0.42	0.67	−0.48	0.64
	Recessive model	2842 / 3333	**1.18**	**1.03–1.35 (0.01)**	54	0.03	1.24	0.21	2.34	0.05
	Allelic model	2842 / 3333	**1.13**	**1.04–1.24 (≤0.001)**	44.6	0.07	0.42	0.67	0.47	0.65
	CC vs. TT	2842 / 3333	1.27	0.97–1.65 (0.08)	42.3	0.09	0.99	0.32	0.97	0.36
	CT vs. TT	2842 / 3333	1.08	0.92–1.26 (0.33)	4.5	0.39	−0.42	0.67	−0.64	0.54
**Continent subgroups**										
**Asia**	Dominant model	3634 / 5371	1.10	0.93–1.130 (0.25)	29.3	0.13	0.81	0.41	1.44	0.17
	Recessive model	3634 / 5371	**1.14**	**1.02–1.26 (0.01)**	0	0.56	0.85	0.39	1.24	0.24
	Allelic model	3634 / 5371	**1.12**	**1.03–1.21 (≤0.001)**	34.9	0.08	2.31	0.02	2.70	0.01
	CC vs. TT	3634 / 5371	1.08	0.87–1.33 (0.49)	0	0.77	0	1	0.09	0.93
	CT vs. TT	3634 / 5371	1.02	0.89–1.18 (0.76)	29.6	0.12	0.45	0.65	0.93	0.36
**Europe**	Dominant model	1129 / 1008	**1.23**	**1.01–1.50 (0.03)**	48.3	0.12	−0.68	0.49	−0.64	0.58
	Recessive model	1129 / 1008	**2.94**	**1.54–5.62 (≤0.001)**	0	0.95	−0.52	0.60	−0.85	0.55
	Allelic model	1129 / 1008	**1.30**	**1.10–1.54 (≤0.001)**	31.2	0.22	0	1	−0.68	0.56
	CC vs. TT	1129 / 1008	**3**	**1.56–5.76 (≤0.001)**	0	0.87	−0.52	0.60	−1.20	0.44
	CT vs. TT	1129 / 1008	1.13	0.92–1.38 (0.24)	52.8	0.09	−0.68	0.49	−0.59	0.61
**America**	Dominant model	1181 / 1278	**1.26**	**1.05–1.51(≤0.001)**	0	0.76	0.45	0.65	0.54	0.61
	Recessive model	1181 / 1278	1.16	0.88–1.52 (0.29)	0	0.89	2.55	0.01	6.60	0.06
	Allelic model	1181 / 1278	**1.17**	**1.03–1.33 (0.01)**	0	0.55	1.65	0.09	2.71	0.04
	CC vs. TT	1181 / 1278	1.26	0.93–1.71 (0.14)	0	0.84	2.25	0.02	3.17	0.05
	CT vs. TT	1181 / 1278	**1.23**	**1.02–1.49 (0.03)**	0	0.85	0.45	0.65	0.18	0.86
**Ethnicity subgroups**										
**Caucasian**	Dominant model	5881 / 7710	**1.15**	**1.04–1.28 (0.008)**	30.7	0.08	0.78	0.43	0.69	0.50
	Recessive model	5881 / 7710	**1.17**	**1.06–1.30 (0.002)**	6.7	0.3	1.98	0.04	2.44	0.02
	Allelic model	5881 / 7710	**1.14**	**1.07–1.22 (≤0.001)**	35.7	0.04	1.87	0.06	1.79	0.08
	CC vs. TT	5881 / 7710	**1.23**	**1.01–1.49 (0.03)**	4.6	0.4	1.14	0.25	0.72	0.48
	CT vs. TT	5881 / 7710	1.1	0.98–1.22 (0.09)	30.6	0.08	−0.68	0.49	−0.59	0.61
**African-American**	Dominant model	706 / 698	1.14	0.89–1.45 (0.29)	0	0.76	0.45	0.65	0.54	0.61
	Recessive model	706 / 698	1.08	0.80–1.46 (0.6)	0	0.87	2.55	0.01	6.60	0.06
	Allelic model	706 / 698	1.08	0.93–1.27 (0.32)	0	0.74	1.65	0.09	2.71	0.04
	CC vs. TT	706 / 698	1.16	0.82–1.62 (0.4)	0	0.79	2.25	0.02	3.17	0.05
	CT vs. TT	706 / 698	1.13	0.87–1.45 (0.35)	0	0.8	0.45	0.65	0.18	0.86

### Subgroup analysis

We categorized studies into different subgroups on the basis of age, continent and ethnicity. The results of pooled ORs, heterogeneity tests and publication bias tests for different analysis models are reported in Table 3**.**

### Subgroup analysis by age

**Fig. 3:**
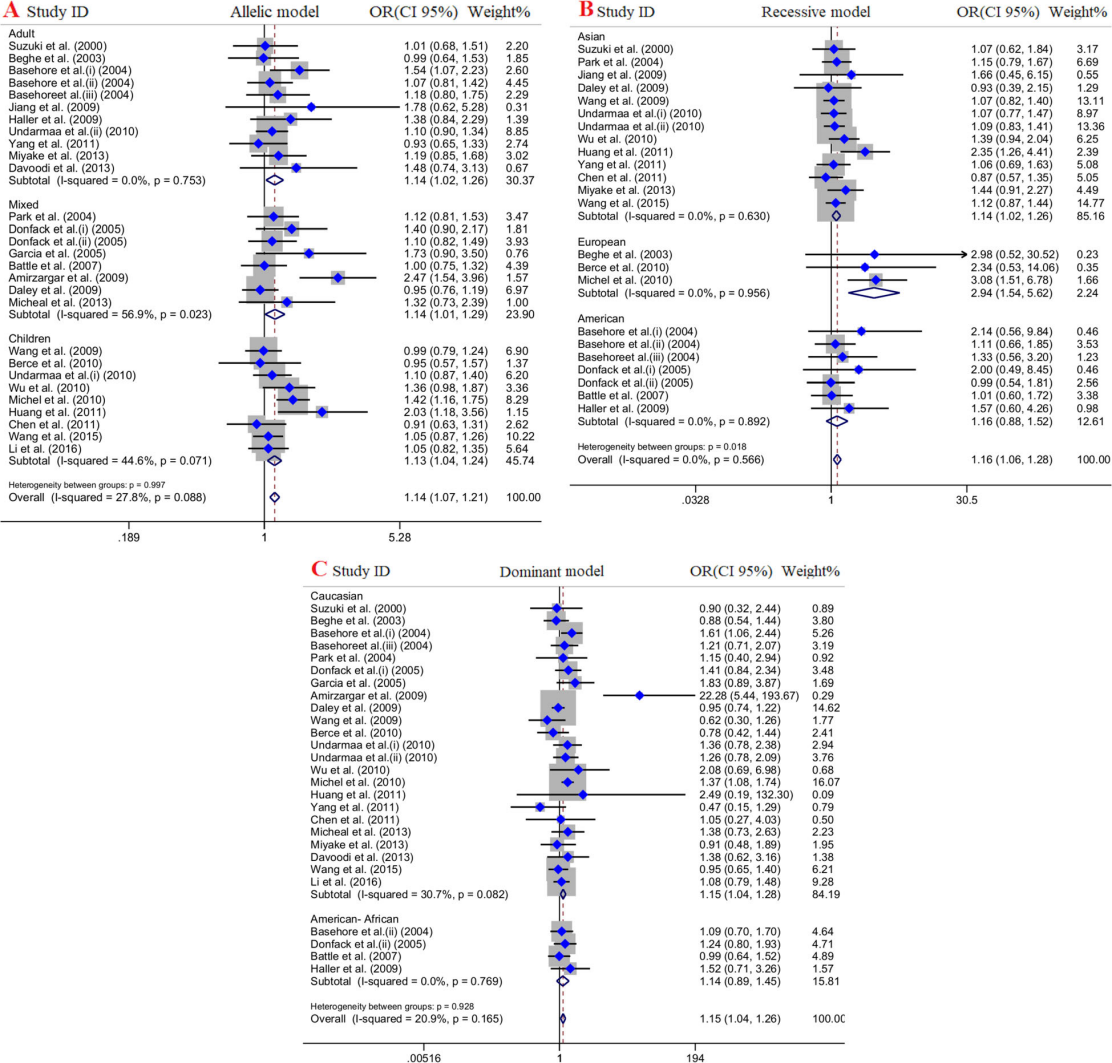
Pooled odds ratio (OR) and 95% confidence interval of individual studies and pooled data for the association between IL-4 C33T gene polymorphism and asthma risk in different subgroup analysis: **a** allelic model, **b** recessive model, **c** dominant model

In this group, we stratified included publications into three groups including: adult (8 articles), children (7 articles) and mixed (cover both ranges; 9 articles). Overall, the results rejected significant association between IL-4 C33T polymorphism and risk of asthma in different age group except for allelic model [adults (OR = 1.14, 95% CI = 1.02–1.26, *P* = 0.02, FEM), mixed (OR = 1.14, 95% CI = 1.01–1.29, *P* = 0.03, REM), children (OR = 1.13, 95% CI = 1.04–1.24, *P* = ≤0.001, FEM)] and recessive model (just in children (OR = 1.18, 95% CI = 1.03–1.35, *P* = 0.01, REM)) Fig. 3.

### Subgroup analysis by continent

Our included studies performed in Asia (15 articles), Europe (4 articles), America (4 articles), and Oceania (1 article). Since there was only one study for Oceania, we exclude it. The final findings indicated strong significant association between IL-4 C33T polymorphism and asthma risk in European population across dominant model (OR = 1.23, 95% CI = 1.01–1.50, *P* = 0.03, FEM), recessive model (OR = 2.94, 95% CI = 1.54–5.62, *P* = ≤0.001, FEM), allelic model (OR = 1.30, 95% CI = 1.10–1.54, *P* = ≤0.001, FEM) and CC vs. TT (OR = 3, 95% CI = 1.56–5.76, *P* = ≤0.001, FEM). Moreover, there was a significant association between IL-4 C33T polymorphism and risk of asthma in American population under dominant model (OR = 1.26, 95% CI = 1.05–1.51, *P* = ≤0.001, FEM), allelic model (OR = 1.17, 95% CI = 1.03–1.33, *P* = 0.01, FEM), and CT vs. TT model (OR = 1.23, 95% CI = 1.02–1.49, *P* = 0.03, FEM). Eventually, Significant positive association was revealed in Asians just in recessive model (OR = 1.14, 95% CI = 1.02–1.26, *P* = 0.01, FEM), and allelic model (OR = 1.12, 95% CI = 1.03–1.21, *P* = ≤0.001, FEM) Fig. 3.

### Subgroup analysis by ethnicity

Finally, we stratified eligible articles according ethnicity including Caucasians (20 articles), and African-Americans (4 articles). The results showed significant association between IL-4 SNP (C33T) and asthma risk in Caucasians under dominant model (OR = 1.15, 95% CI = 1.04–1.28, *P* = 0.008, FEM), recessive model (OR = 1.17, 95% CI = 1.06–1.30, *P* = 0.002, FEM), allelic model (OR = 1.14, 95% CI = 1.07–1.22, *P* = ≤0.001, FEM),and CC vs. TT model (OR = 1.23, 95% CI = 1.01–1.49, *P* = 0.03, FEM) but not CT vs. TT model (OR = 1.1, 95% CI = 0.98–1.22, *P* = 0.09, FEM). However, there was no significant association between IL-4 C33T polymorphism and risk of asthma in American-African population across all genotype models Fig. 3.

### Evaluation of heterogeneity

No significant heterogeneity was detected for IL-4 C33T polymorphism neither in overall population nor subgroup analysis, therefore we did not perform mete-regression analysis for possible parameters (Table 3).

### Sensitivity analysis and publication bias

**Fig. 4:**
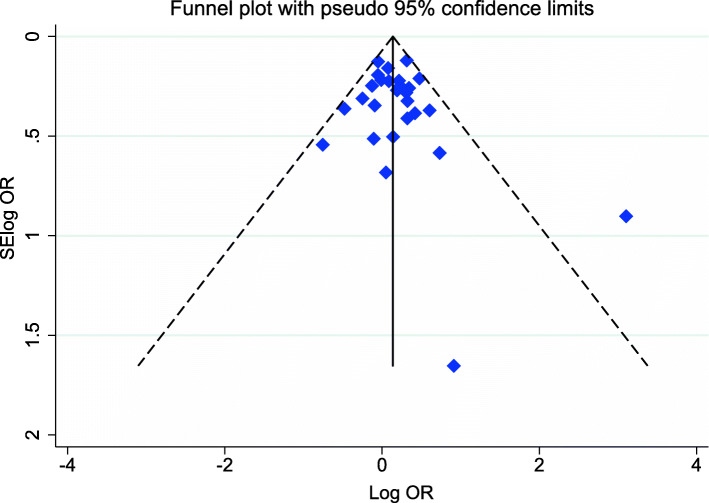
Begg’s funnel plot for publication bias test. Dominant model IL-4 C33T. Each point represents a separate study for the indicated association

**Fig. 5:**
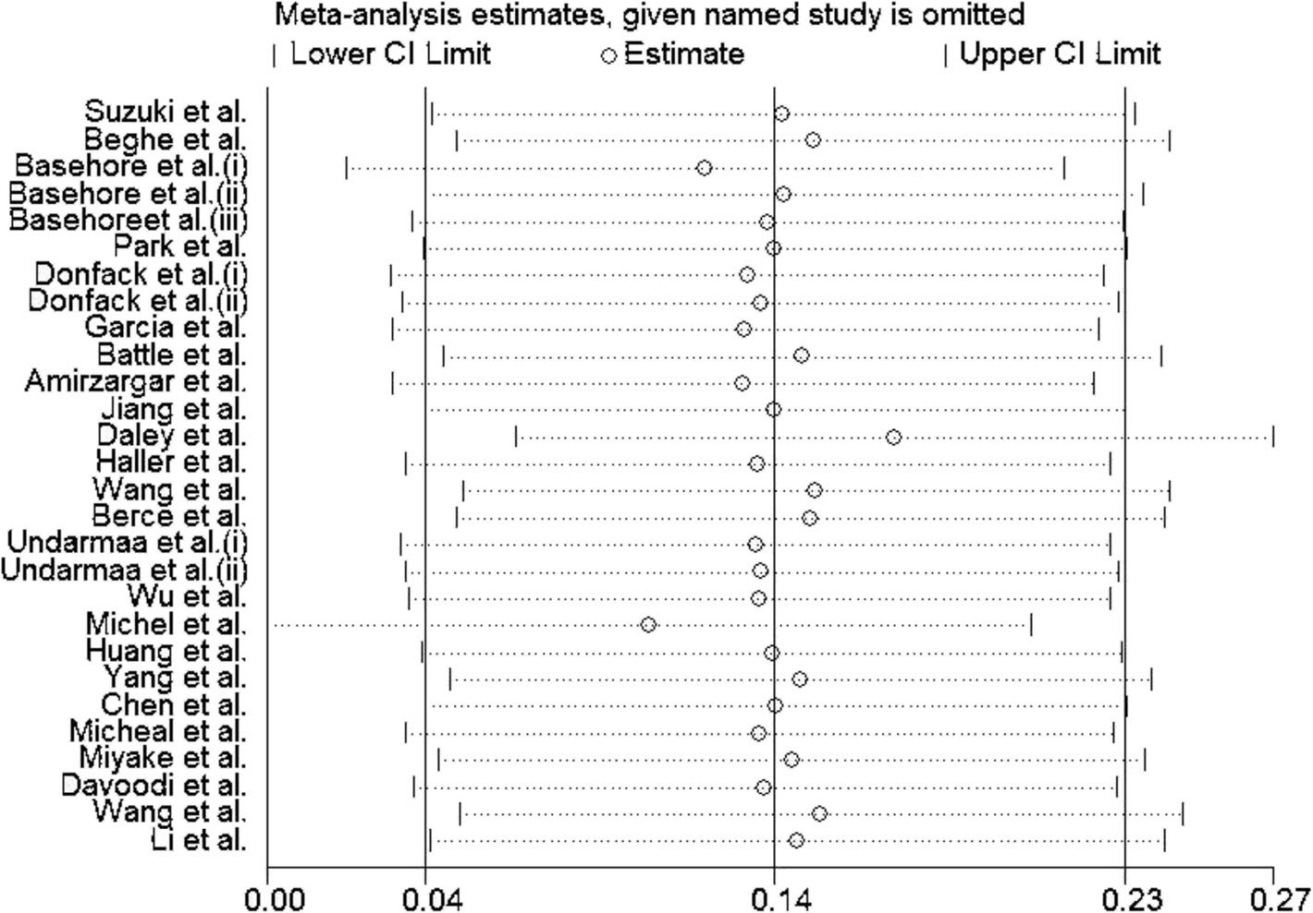
Sensitivity analysis in present meta-analysis investigates the single nucleotide polymorphisms of IL-4 C33T contribute to risk for asthma

Begg’s and Egger’s tests were performed to estimate the publication biases of studies. As showed in Table 3 no evidence of publication bias was detected in overall populations and subgroup analysis. Also, symmetric shape of Begg’s funnel plot confirm this finding Fig. 4. Moreover, the impact of individual study on pooled OR was evaluated by sensitivity analysis, which confirmed stability of our results Fig. 5.

## Discussion

The cytokine IL-4 act as a key player in the development and pathogenesis of allergic inflammation [37] and atopy [38] through the induction of the heavy chain isotype switching, secretion of IgE antibody (IgE synthesis) by B cells, functioning as a growth factor for Th2 cells [37]. The IL-4 promotes IgE-dependent immune responses as it induces overexpression of IgE receptors on the surface of various immune cells: FcεRI on basophils and mast cells; and FcεRII (CD23) on mononuclear phagocytic cells and B lymphocytes [39]. The IL-4 tilts the immune response to anti-inflammatory, inhibiting macrophages pro-inflammatory effect and downregulating secretion of pro-inflammatory cytokines [40]. The IL-4 critically, initiate immediate allergic responses by triggering IgE-mediated mast cell activation [41]. The IL-4 plays a pivotal role in the priming of naïve T cell towards Th2 differentiation as well as exacerbate allergic inflammation through induction of vascular adhesion molecule 1 (VCAM-1) that recruit leukocytes and promote their survival [39]. The IL-4 induce airway remodeling encountered in asthma by its role in the proliferation of bronchial fibroblasts, myofibroblasts, and airway smooth muscles [38].

At the turn of the millennium genetic polymorphisms of the IL-4 gene in the development and maintenance of asthma have drawn increasing consideration. Modulation of the immune system is the common denominator in IL-4 polymorphisms [40]. Suzuki and coworkers found a single nucleotide polymorphism of C replacement of T at position 33 bp of exon 1 (C33T) of the IL-4 proximal promoter region [42]. Asthmatic patients with C33T have higher serum level of IL-4 and IgE [43]. Anovazzi and colleagues studied IL-4 haplotypes and reported that the studied haplotypes induce an opposing immune response, as well they recorded minimal functional activity in polymorphisms involving the promoter region [40]. An increasing body of evidence has demonstrated that C33T of the IL-4 gene untranslated region (UTR) of chromosome 5q was associated with elevated serum IgE levels and the risk of asthma [44, 45]. However, this association remains inconclusive. If this is indeed the case, a meta-analysis with big sample size, sufficient statistical power, and subgroup analysis was needed.

Our current meta-analysis composed of 24 publications (28 studies) involving 6587 cases and 8408 controls, we systematically assessed the relationship between IL-4 C33T polymorphism and asthma susceptibility. Cumulatively, the result illustrated IL-4 C33T polymorphism as a risk factor in the pathogenesis of asthma. The result indicated that the presence of T allele across different genetic models increased asthma risk by 10 to 21%. In the subgroup analysis by age, the results rejected the significant association between IL-4 C33T polymorphism and risk of asthma in different age groups except for allelic model, which highlighted the predisposing role of the T allele for the asthma risk in all three age groups. Subgroup analysis by continent revealed a significant association between IL-4 C33T polymorphism and asthma risk in the European population. In the Asian population, there was a significant association between IL-4 C33T polymorphism and the risk of asthma under recessive and allelic models. In contrast, the American population showed a significant association under dominant and allelic models.

Additionally, subgroup analysis was conducted according to ethnicity; the results showed a significant association between IL-4 C33T polymorphism and asthma risk in Caucasians under all models except CT vs TT model. While, no significant association between IL-4 C33T polymorphism and the risk of asthma in the American-African population were detected.

It should be noted that our results are not in agreement with those of Liu et al. [46] meta-analysis on the role of IL-4 C33T polymorphism and asthma. They suggested a significant association between whites and Asians. While they reported a significant association between the IL-4 C33T polymorphism and asthma risk in the overall population, they did not find a significant association among atopic and non-atopic asthma patients in subgroup analysis. Furthermore, in contrast to our meta-analysis, in the subgroup analysis by age, they reported an increased risk of asthma among children but not in the adult. Finally, while they reported evidence of publication bias, we identified no evidence of publication bias for the overall population and subgroup analysis under all genetic models.

The main reason for these discrepancies raised could be from the fact that Liu and colleagues included 18 studies with 5523 cases and 5618 controls. However, our meta-analysis encompasses 28 studies including 6587 cases and 8408 controls from different ethnicities and continents.

The C33T single nucleotide polymorphism is detected on the 5′ untranslated regions (UTR) of the IL-4 gene [42]. The 5′ UTRs region of mRNA may contain many gene regulatory elements (GRE) that regulate the localization, translation and degradation of transcripts [47]. In the eukaryotic mRNAs, the 5′ UTRs regulate both cap-dependent and cap-independent translation initiation of mRNA [48]. Researchers revealed a relationship between IL-4 C33T polymorphism and elevated serum IgE levels in a group of the Japanese population [49]. While the exact mechanisms by which the IL-4 C33T allele modulates the gene expression of the IL-4 remain elusive, it has been suggested that this variation may influence the stability of mRNA, as well as transcriptional or translational efficiency of the IL-4 gene, highlighting that the 5′ UTR may involve many cis-acting elements [47, 50–52].

Heterogeneity and publication bias, which may affect the results of meta-analyses, should always be considered. The result of this study did not show significant heterogeneity. Moreover, there was no significant publication bias in the overall population and subgroup analysis under all genetic models. Consequently, heterogeneity and publication bias did not appear to have inclined the results. Sensitivity analyses were also performed. There was a little variation of the estimates after exclusion of a single study and the significance of the pooled ORs was not affected proposing the consistency of this result.

The current study had some limitations. First, most included articles were from the Asia continent with Caucasian race and there was no study from Africans; accordingly, the results of this meta-analysis may not be appropriate to Africans. Second, in some studies, the diagnostic criteria and asthma phenotype were not clearly determined; while the asthma diagnostic criteria were primarily based on physical examination, clinical history, and pulmonary function tests (PFT), there did exist a little dissimilarity among studies. Third, the overall results were based on unadjusted estimates; a more precise evaluation should be accompanied when all singular raw data are accessible, which would facilitate the adjustment by other potential co-variants such as; age, gender, obesity, environmental factors, smoking status, and other lifestyles. Fourth, due to a lack of extractable data, we failed to address gene-environment and gene-gene interactions. In contrast to these limitations, two main strengths of our meta-analysis include; Firstly, a large number of patients and the healthy individuals were pooled from various studies, which considerably augment the statistical power of the meta-analysis. Secondly, no evidence of publication biases was identified, representing that the whole collected data may be unbiased.

## Conclusion

Taken together, this study suggests that IL-4 C33T polymorphism potentially acts as a risk factor for asthma in different ethnicities and age groups. Nevertheless, large sample studies from different continents and races with homogeneous asthmatic patients and well-matched healthy subjects are still needed. Furthermore, gene-environment and gene-gene interactions should also be regarded in future studies. With taking these factors into account in future studies, it would ultimately lead to our comprehensive and better understanding of the association between the IL-4 C33T polymorphism and asthma susceptibility.

## Data Availability

All data generated or analyzed during this study are included in this published article.

## Abbreviations

IL-4: interleukin 4
SNP: Single nucleotide polymorphisms
PRISMA: Preferred Reporting Items for Systematic reviews and Meta-Analyses
NOS: Newcastle-Ottawa Scale
